# Care dependency and postoperative recovery after total knee arthroplasty: a cross-sectional study

**DOI:** 10.3389/fpubh.2025.1664154

**Published:** 2025-11-14

**Authors:** Esra Nacakçıoğlu, Hande Cengiz Açıl

**Affiliations:** 1 Yozgat City Hospital, Yozgat, Türkiye; 2 Department of Surgical Nursing, Faculty of Health Sciences, Sakarya University, Sakarya, Türkiye

**Keywords:** care dependency, recovery, total knee arthroplasty, knee replacement, postoperative care

## Abstract

**Background:**

This study aimed to determine the effect of care dependency levels on postoperative recovery in patients undergoing total knee arthroplasty (TKA). Patients who undergo TKA may face difficulties in performing daily living activities after surgery. Care dependency is a critical factor that can negatively impact the recovery process. Therefore, evaluating its influence is important for improving postoperative outcomes.

**Methods:**

This cross-sectional and descriptive study was conducted with 100 patients who underwent TKA between February 1, 2023, and February 1, 2024, and met the inclusion criteria. Data were collected using a Personal Information Form, the Care Dependency Scale (CDS), and the Postoperative Recovery Index (PoRI). The CDS assessed the patients’ levels of dependency, while the PoRI evaluated their recovery status. The data were analyzed using descriptive statistics and correlation analysis.

**Results:**

The mean age of participants was 63.90 ± 7.87 years; 75% were female, and 79% were married. Among them, 55% were primary school graduates and 49% were unemployed. Right knee surgery was performed in 50% of the cases; 53% had chronic illnesses, and 43% had a prior surgical history. The mean CDS score was 58.43 ± 9.87, and the mean PoRI score was 2.82 ± 0.68. Based on CDS scores, 46% of the patients were classified as “dependent.” A significant correlation was found between increased age and greater difficulty in both care dependency and recovery. However, gender did not have a statistically significant effect on recovery.

**Conclusion:**

The study revealed that higher care dependency levels negatively affect postoperative recovery in TKA patients. Recovery was particularly more challenging for older individuals, those with chronic conditions, lower education levels, and prior surgeries. These findings highlight the need for individualized nursing and care plans, especially for patients with high care dependency.

## Introduction

As the world population is getting older, musculoskeletal diseases are also increasing. Osteoarthritis (OA) is one of the most common musculoskeletal diseases in the older adults and negatively affects the quality of life of older adults individuals. Knee osteoarthritis is a chronic, degenerative joint disease that causes loss of cartilage tissue and new bone formation on joint surfaces (1). The most common findings in knee osteoarthritis are decreased physical function, pain and stiffness in the joints. The goal in the treatment of knee osteoarthritis is to improve the quality of life by controlling pain and other symptoms and to enable the patient to fulfill and maintain physical functions (1). Osteoarthritis is the most common type of arthritis leading to total knee arthroplasty (TKA). Total knee arthroplasty (TKA) is a surgical procedure to replace the knee joint with a prosthetic implant due to severe arthritis or injury (2). Care dependency is defined as the patient’s need for support provided by specialists, a decrease in the level of fulfillment of self-care needs, and a certain level of care (refers to the support required for daily living activities, including assistance with mobility, personal hygiene, and medication management, depending on the patient’s dependency level) according to the dependency status (3). After total knee arthroplasty (TKA), patients may need to use assistive devices such as crutches, wheelchairs and walkers. During this period, the patient is more dependent on his/her relatives and the nurse. Patients undergoing TKA surgery may experience physical dependence due to pain, mobility limitation and loss of independence in the postoperative period. This physical dependence may negatively affect not only the patients’ ability to maintain their activities of daily living but also their psychological state. Patients’ limited mobility and dependence on others can lead to psychological problems such as anxiety, depression and loss of motivation. These conditions can negatively affect patients’ compliance with rehabilitation and overall quality of life during the recovery process, leading to prolonged recovery time. On the other hand, these effects can be minimized with appropriate psychosocial support and nursing care, thus contributing positively to the recovery process. Nursing care requires a comprehensive approach to address the physical, psychological and social needs of patients undergoing TKA. In particular, mobilization support should be provided to increase the level of independence of patients in the postoperative period, pain management should be performed, and patients should be directed to individual and group therapies to improve their psychological status (1, 4). Ünal & Gürhan (5) emphasize the importance of addressing patients’ cognitive, physical, and social needs during postoperative care. However, it is important to note that the extent of nursing care may vary depending on healthcare systems and cultural contexts. For instance, in outpatient-based procedures commonly seen in the United States, nursing care might focus on specific recovery milestones rather than comprehensive care. It is thought that determining the care dependency levels of the patients will be beneficial for the patients in the process of increasing the patient’s well-being, ensuring recovery and preventing complications related to the surgery after TKA. Although limited studies have examined the relationship between care dependency and postoperative recovery, several studies have evaluated recovery outcomes after total knee arthroplasty using validated instruments such as QoR-15, QoR-40, and PoRI. Postoperative care dependency significantly affects recovery outcomes in patients undergoing total knee arthroplasty. Understanding and addressing care dependency levels can lead to personalized nursing strategies that enhance recovery trajectories. This study was planned to determine the levels of care dependency in patients who underwent total knee arthroplasty and to determine the effect of care dependency levels on recovery.

## Method

### Design and sample

This study employed a cross-sectional and descriptive design. Data were collected using standardized tools administered at multiple points during the postoperative recovery period. Statistical analyses, including descriptive and inferential tests, were used to assess correlations between care dependency levels and recovery outcomes. The study population consisted of patients who underwent total knee replacement between 01.02.2023 and 01.02.2024 in the orthopedic clinics of Sakarya University Training and Research Hospital, and the sample consisted of 100 patients who met the research criteria and agreed to participate in the study. All patients underwent total knee arthroplasty using the same surgical approach and followed a standardized postoperative physical therapy protocol to ensure consistency in outcomes. All procedures were performed on an inpatient basis, with patients being discharged within 3–5 days based on their recovery progress. Factors such as age, comorbidities, pre-existing mobility limitations, and psychosocial support were considered as potential confounders in the analysis. Each patient was evaluated once during the postoperative period (between days 1 and 4), and the specific day of assessment was recorded and included in statistical analyses as a covariate to control for confounding effects related to recovery timing. A convenience sampling method was used, including all patients who met the eligibility criteria and agreed to participate. The participation rate was 100%.

The research universe consisted of patients who underwent total knee replacement surgery in the orthopedic clinics of Sakarya University Training and Research Hospital between 01.02.2023–01.02.2024, and the sample consisted of 100 patients who met the research criteria and agreed to participate in the study. The participants who agreed to participate in the study were given the necessary information and had their written consent signed by the Informed Consent Form. The *a priori* power analysis was performed using G*Power 3.1.9.7 based on previous studies reporting a large correlation between care dependency and recovery (effect size *r* = 0.75). With *α* = 0.05 and *n* = 100, the calculated power was 0.99, indicating sufficient sample size.

Individuals to be included in the sample were selected by considering the following criteria:

- Having undergone total knee arthroplasty (TKA),
- Being 18 years of age and older,
- Being conscious and oriented to place and time,
- Not having hearing and speech problems.

Exclusion Criteria from the Sample Patients;

- Refusing to participate in the study,
- Not being in the post-operative period,
- Revision TKA,
- Presence of severe postoperative complications or cognitive disorders
- Inability to communicate in Turkish, patients were not included in the study.

### Surgical and rehabilitation protocol

- All total knee arthroplasty (TKA) procedures were performed using a standardized surgical technique.
- All operations were performed under spinal anesthesia with the same surgical team to ensure consistency.
- Postoperative pain control followed a multimodal regimen consisting of paracetamol and nonsteroidal anti-inflammatory drugs (NSAIDs).
- Rehabilitation was standardized: all patients began inpatient physiotherapy within 24 h after surgery, including active and passive knee exercises, gradual mobilization, and gait training under supervision.
- This standardization ensured comparable surgical and rehabilitation conditions across all participants, minimizing procedural variability.

### Ethıcal aspects of the research

Before starting the study, Ethics Committee Approval (Ethics Committee Approval Number: E-71522473-050.01.04-300145-331) was obtained from Sakarya University Faculty of Medicine Clinical Research Ethics Committee and institutional permissions were obtained from the hospital where the study was conducted. All patients were adults and provided written informed consent themselves.

### Data collection

Personal Information Form, Care Dependency Scale and Postoperative Recovery Index were used to collect the data.

### Personal information form

It consists of questions about age, gender, educational status, marital status, occupation, clinic, clinical diagnosis, any chronic disease status, medication used continuously, history of previous surgical intervention, preoperative hospital stay, duration of surgery, type of anesthesia, duration of anesthesia, postoperative day, time of first mobilization and dependency level.

### Care dependency scale

The care dependency scale was developed by Dijkstra et al. (6) and is a 15-item scale created to assess the care dependency status of patients based on Virginia Henderson’s basic human needs. In the scale, which was validated and reliably validated in Turkish by Yönt et al. (7), the cronbach alpha coefficient was found to be 0.91 and the scale was reduced to 17 items. In this study, the Cronbach alpha coefficient was found to be 0.91. The scale, which determines the dependency levels of individuals and is graded with a 5-point Likert-type scoring (the scoring of the scale is 1, completely dependent; 5, completely independent), consists of a total of 17 items including activities of daily living. A minimum score of 17 points and a maximum score of 85 points can be obtained from the scale. A high scale score indicates that the patient is independent in meeting care needs, while a low scale score indicates that the patient is dependent on others in meeting care needs (7).

### Postoperative recovery index

Developed in 2012 by Butler et al., the Turkish validity and reliability study of the Postoperative Recovery Index was conducted by Cengiz &Aygin (8). The index consists of 25 items and 5 sub-dimensions. The sub-dimensions of the index consist of psychological symptoms, physical activities, general symptoms, bowel symptoms and appetite symptoms. When determining the sub-dimension score, the scores of the relevant items are summed and their arithmetic averages are taken. For the total score, all items are summed and the arithmetic mean is taken. Higher scores on the index reflect more difficulty in postoperative recovery, while lower scores indicate that postoperative recovery is easier. In Cengiz and Aygin’s study, the Cronbach’s alpha value of the PoRI was determined as 0.96 (8).

### Statistical analysis

The study was performed with 100 patients. The data were completed using IBM SPSS Statistics 23 program. While analyzing the study data, frequency distribution (number, percentage) was used for categorical variables and descriptive statistics (mean, standard deviation, minimum, maximum) were used for numerical variables. The difference between two groups was analyzed by independent sample t test, and the difference between more than two groups was analyzed by one-way analysis of variance (One Way ANOVA). As a result of the “one-way analysis of variance” (ANOVA), Levene’s test was performed for homogeneity of variance, and then the group or groups from which the difference originated were checked with the “multiple comparison test.” To control for the risk of type I error due to multiple testing, Bonferroni correction was applied to all pairwise and *post-hoc* comparisons, and Tamhane’s T2 test was used to examine the difference between groups in variables that did not provide homogeneity of variance.

**The PORI total score was identified as the primary outcome variable.** Pearson correlation and multivariate linear regression analyses were performed to identify factors associated with recovery, including potential confounders: **age, sex, education, comorbidity, postoperative day, and time to mobilization.**

Pearson correlation analysis was used to examine the relationship between the scales and multiple linear regression analysis was used to examine the factors affecting the scale scores. Cronbach’s alpha value was used for scale reliability. Significance was set at *p* < 0.05 with 95% confidence intervals.

## Results

### Findings related to demographic characteristics of patients

The mean age of the patients was 63.90 ± 7.87 years, 75.0% were female, 79.0% were married, 55.0% were primary school graduates and 49.0% were not working. While 50% of the patients had right TKA, 53% had chronic diseases. The rate of previous surgery was 43 and 18% were on the first postoperative day. The first mobilization time of 84% of the patients was on the first day and the dependency level of 46% was found to be dependent. The care dependency scale score was 58.43 ± 9.87 and the total postoperative recovery index score (PoRI) was 2.82 ± 0.68, while the psychological symptoms sub-dimension score was 2.27 ± 0.78, the physical activities sub-dimension score was 4.16 ± 0.70, the general symptoms sub-dimension score was 2.00 ± 0.88, the bowel symptoms sub-dimension score was 1.60 ± 1.01 and the appetite symptoms sub-dimension score was 3.03 ± 1.16.

While there was no statistically significant difference between gender and care dependency scale score (*p* > 0.05), there was a statistically significant difference between marital status, educational status and occupations in terms of care dependency scale score (*p* < 0.05; Table 1). Bonferroni corrections were applied in all multiple group comparisons to control for type I error inflation.

**Table 1:** Examination of the relationship between care dependency scale score and demographic characteristics.

Demographic characteristics		Care dependency scale	Test	*p*
		Mean ± sd		
Gender	Male	57.12 ± 10.23	−0.764t	0.446
	Female	58.87 ± 9.78		
Marital status	Married	60.01 ± 9.87	3,820t	0.000*
	Single	52.48 ± 7.47		
Educational background	Illiterate	53.00 ± 6.50b	8,335F	0.000*
	Primary education	58.02 ± 9.77b		
	High school	69.27 ± 9.64a		
	Bachelor’s degree and above	60.33 ± 7.92		
Job	Working	62.96 ± 10.38a	4,122F	0.019*
	Not working	57.33 ± 9.47		
	Retired	55.88 ± 8.89b		

a,b shows the mean differences between groups (a: highest mean). F, One-way ANOVA test; *t*, Independent sample *t* test. *p < 0.05.

There was no statistically significant difference between chronic disease status and previous surgical operation status in terms of care dependency scale score (*p* > 0.05), whereas there was a statistically significant difference between clinical diagnoses, day of surgery, time of first mobilization and dependency levels in terms of care dependency scale score (*p* < 0.05; Table 2).

**Table 2:** Examination of the relationship between care dependency scale score and health history characteristics.

Health history characteristics		Care dependency scale	Test	*p*
		Mean ± sd		
Operated side	Right TKA	55.98 ± 9.30	−2,713t	0.008*
	Left TKA	61.25 ± 9.93		
Chronic disease condition	Present	56.89 ± 9.27	−1,675t	0.097
	Absent	60.17 ± 10.33		
Previous surgery	Yes	57.93 ± 9.13	−0.438t	0.662
	No	58.81 ± 10.46		
Postoperative day	1 day	52.44 ± 5.15b	4,800F	0.004*
	2 days	59.58 ± 10.23		
	3 days	57.19 ± 9.29		
	4th day and above	63.68 ± 10.64a		
First mobilization time	0–12.hour	53.15 ± 4.14	−3,816t	0.000*
	1 day	59.22 ± 10.25		
Dependency level	Dependent	51.72 ± 5.95c	77.618F	0.000*
	semi-dependent	59.92 ± 6.90b		
	Independent	73.35 ± 5.02a		

a,b,c shows the average differences between groups (a: highest average). F, One-way ANOVA test; *t*, Independent sample *t* test. *p < 0.05.

There is a statistically significant difference between marital statuses in terms of the appetite symptoms sub-dimension score (*p* < 0.05; Table 3). There is a statistically significant difference (*p* < 0.05) between the educational status in terms of postoperative recovery index scale and psychological symptoms, physical activities, and appetite symptoms sub-dimension scores (Table 3). There was a statistically significant difference (*p* < 0.05) between the occupations in terms of postoperative recovery index scale and physical activities sub-dimension scores (Table 3). There was no statistically significant difference between genders in terms of postoperative recovery index scale and sub-dimension scores (*p* > 0.05; Table 3).

**Table 3:** Examining the relationship between postoperative recovery index score and demographic characteristics.

Demographic characteristics	Psychological symptoms	Physical activities	General symptoms	Bowel symptoms	Appetite symptoms	PoRI
	Mean ± sd	Mean ± sd	Mean ± sd	Mean ± sd	Mean ± sd	Mean ± sd
Gender						
Male	2.51 ± 0.78	4.27 ± 0.61	2.11 ± 0.88	1.61 ± 0.89	3.21 ± 1.07	2.94 ± 0.57
Female	2.19 ± 0.76	4.12 ± 0.73	1.96 ± 0.89	1.60 ± 1.05	2.98 ± 1.19	2.78 ± 0.71
**t /p**	1.788/0.077	0.876/0.383	0.734/0.464	0.023/0.982	0.870/0.386	1.014/0.313
Marital status						
Married	2.25 ± 0.78	4.10 ± 0.70	1.98 ± 0.95	1.61 ± 1.03	2.90 ± 1.17	2.78 ± 0.70
Single	2.37 ± 0.78	4.37 ± 0.66	2.06 ± 0.55	1.57 ± 0.93	3.55 ± 1.00	2.99 ± 0.56
**t /p**	−0.640/0.524	−1.562/0.122	−0.486/0.629	0.166/0.868	−2.330/0.022 *	−1.286/0.202
Educational background						
Illiterate	2.65 ± 0.75a	4.51 ± 0.36a	2.19 ± 0.79	1.36 ± 0.90	3.53 ± 0.95a	3.06 ± 0.54a
Primary education	2.31 ± 0.74a	4.19 ± 0.70a	2.06 ± 0.97	1.77 ± 1.11	3.06 ± 1.08a	2.88 ± 0.66a
High school	1.50 ± 0.32b	3.48 ± 0.72b	1.41 ± 0.44	1.09 ± 0.30	2.07 ± 1.24b	2.13 ± 0.53b
Bachelor’s degree and above	2.13 ± 0.82	3.99 ± 0.73	1.90 ± 0.75	1.75 ± 0.97	2.90 ± 1.32	2.73 ± 0.72
**f/p**	6.460/0.000 *	6.570/0.000 *	2.213/0.092	2.023/0.116	4.380/0.006 *	5.671/0.001 *
Job						
Working	1.97 ± 0.78	3.75 ± 0.78b	1.70 ± 0.70	1.52 ± 0.78	2.60 ± 1.22	2.51 ± 0.67b
Not working	2.34 ± 0.77	4.25 ± 0.67a	2.05 ± 1.00	1.78 ± 1.19	3.18 ± 1.17	2.93 ± 0.73a
Retired	2.45 ± 0.73	4.40 ± 0.48a	2.21 ± 0.75	1.35 ± 0.76	3.21 ± 0.99	2.94 ± 0.47a
**f/p**	2.924/0.058	6.953/0.002 *	2.318/0.104	1.654/0.197	2.604/0.079	3.981/0.022 *

a,b shows the mean differences between groups (a: highest mean). F, One-way ANOVA test; *t*, Independent sample *t* test. *p < 0.05. It was stated that the bold expression in all of them was *p* < 0.005.

There is no statistically significant difference between clinical diagnoses in terms of postoperative recovery index scale and subscale scores (*p* > 0.05; Table 4). There is a statistically significant difference between chronic disease states in terms of general symptoms sub-dimension score (*p* < 0.05; Table 4). There is a statistically significant difference (*p* < 0.05) in the general symptoms and bowel symptoms sub-dimension scores between previous surgical operations (Table 4). There is a statistically significant difference (*p* < 0.05) between the days of surgery in terms of postoperative recovery index scale and physical activities, appetite symptoms sub-dimension scores (Table 4). There is a statistically significant difference between the first mobilization times in terms of physical activities subscale score (*p* < 0.05; Table 4). There was a statistically significant difference (*p* < 0.05) between the commitment levels in terms of postoperative recovery index scale and sub-dimension scores (Table 4).

**Table 4:** Examination of the relationship between postoperative recovery index score and health history characteristics.

Health history characteristics	Psychological symptoms	Physical activities	General symptoms	Bowel symptoms	Appetite symptoms	PoRI
	Mean ± sd	Mean ± sd	Mean ± sd	Mean ± sd	Mean ± sd	Mean ± sd
Clinical diagnosis						
Right TKA	2.36 ± 0.82	4.23 ± 0.73	2.14 ± 0.80	1.65 ± 1.11	3.19 ± 1.19	2.91 ± 0.69
Left TKA	2.13 ± 0.68	4.06 ± 0.66	1.84 ± 0.96	1.50 ± 0.87	2.87 ± 1.14	2.70 ± 0.64
**t /p**	1.500/0.137	1.151/0.253	1.660/0.100	0.734/0.465	1.338/0.184	1.598/0.113
Chronic disease condition						
Present	2.35 ± 0.74	4.25 ± 0.61	2.19 ± 0.98	1.65 ± 1.02	3.09 ± 1.09	2.91 ± 0.62
Absent	2.19 ± 0.81	4.05 ± 0.78	1.78 ± 0.71	1.55 ± 1.00	2.97 ± 1.24	2.72 ± 0.73
**t /p**	1.049/0.297	1.444/0.152	2.397/0.018 *	0.514/0.609	0.541/0.589	1.465/0.146
Previous surgical operation						
Yes	2.34 ± 0.73	4.17 ± 0.66	2.30 ± 1.05	1.89 ± 1.12	3.09 ± 1.18	2.95 ± 0.63
No	2.22 ± 0.81	4.15 ± 0.74	1.77 ± 0.66	1.39 ± 0.86	3.00 ± 1.15	2.72 ± 0.70
**t /p**	0.723/0.471	0.122/0.903	2.944/0.004 *	2.474/0.016 *	0.389/0.698	1.664/0.099
Which day of the surgery?						
1 day	2.51 ± 0.74	4.63 ± 0.31a	2.18 ± 0.79	1.41 ± 0.84	3.39 ± 0.89a	3.06 ± 0.42a
2 days	2.26 ± 0.87	4.13 ± 0.67	2.15 ± 1.01	1.67 ± 1.04	2.99 ± 1.14	2.84 ± 0.72
3 days	2.33 ± 0.70	4.24 ± 0.63a	1.99 ± 0.81	1.87 ± 1.23	3.31 ± 1.21a	2.95 ± 0.70a
4th day and above	1.99 ± 0.69	3.66 ± 0.81b	1.54 ± 0.69	1.27 ± 0.60	2.39 ± 1.16b	2.37 ± 0.58b
**f/p**	1.525/0.213	7.061/0.000 *	2.439/0.069	1.639/0.185	3.241/0.025 *	4.197/0.008 *
First mobilization time						
0-12.hour	2.31 ± 0.65	4.54 ± 0.32	1.88 ± 0.74	1.32 ± 0.68	3.29 ± 0.94	2.91 ± 0.29
1 day	2.27 ± 0.79	4.10 ± 0.72	2.01 ± 0.90	1.65 ± 1.04	3.00 ± 1.19	2.81 ± 0.72
**t /p**	0.175/0.862	3.727/0.001 *	−0.493/0.623	−1.479/0.153	0.844/0.401	0.965/0.340
Dependency level						
Dependent	2.66 ± 0.76a	4.64 ± 0.30a	2.46 ± 0.83a	1.85 ± 1.16a	3.60 ± 0.93a	3.25 ± 0.49a
semi-dependent	2.10 ± 0.56b	4.08 ± 0.49b	1.76 ± 0.74b	1.57 ± 0.92	3.03 ± 1.00b	2.72 ± 0.52b
Independent	1.59 ± 0.62c	3.01 ± 0.38c	1.28 ± 0.61b	1.00 ± 0.00b	1.53 ± 0.53c	1.87 ± 0.20c
**f/p**	17.925/0.000 *	107.006/0.000 *	17,698/0,000 *	4.830/0.010 *	32.190/0.000 *	56.156/0.000 *

a,b,c shows the mean differences between groups (a: highest mean). F, One-way ANOVA test; *t*, Independent sample *t* test. *p < 0.05. It was stated that the bold expression in all of them was *p* < 0.005.

There is a statistically significant negative relationship between the care dependency scale and postoperative recovery index and sub-dimension scores (*p* < 0.05; Table 5).

**Table 5:** Examination of the relationship between the care dependency scale and the postoperative recovery index total score and sub-dimensions.

Care dependency scale		Psychological symptoms	Physical activities	General symptoms	Bowel symptoms	Appetite symptoms	PoRI
Care dependency scale	*r*	−0.509	−0.827	−0.434	−0.318	−0.729	−0.752
	*p*	0.000*	0.000*	0.000*	0.000*	0.000*	0.000*
	N	one hundred	one hundred	one hundred	one hundred	one hundred	one hundred

r, Pearson correlation coefficient. *p < 0.05. It was stated that the bold expression in all of them was *p* < 0.005.

Pearson correlation analysis demonstrated a significant negative correlation between CDS and PORI total and subscale scores (*r* = −0.75, *p* < 0.001). Higher dependency levels were associated with more difficulty in recovery across psychological, physical, and general symptoms.

Marital status, clinical diagnosis and dependency levels have a statistically significant effect on the care dependency scale score. Accordingly, the care dependency scale score of the married patients was 3.193 units higher than the single patients, the care dependency scale score of the left TKA patients was 3.153 units higher than the right TKA patients, the care dependency scale score of the semi-dependent patients was 8.168 units higher than the dependent patients, and the care dependency scale score of the independent patients was 20.780 units higher than the dependent patients (Table 6).

**Table 6:** Findings for examining the factors effective on the care dependency scale score.

The factors	Standardized coefficient		Unstandardized coefficient	*t*	*p*	95.0% CI	
	B.	std. Mistake	Beta			Lower limit	Upper limit
(constant)	50,946	2,888		17,639	0.000	45,212	56,680
marital status	−3.193	1,475	−0.132	−2.165	0.033	−6,121	−0.266
Operated side	3,153	1,099	0.173	2,870	0.005	0.972	5,334
semi-dependent/dependent	8,168	1,291	0.401	6,325	0.000	5,605	10,732
Independent/dependent	20,780	1,675	0.794	12,402	0.000	17,454	24,106

F: 48.206, *p* < 0.001, R^2^: 0.670.

Care dependency scale score and dependency level have a statistically significant effect on postoperative recovery index scale score. Accordingly, when the care dependency scale score increases by 1 unit, the postoperative recovery index scale score decreases by 0.032 units, while the postoperative recovery index scale score of semi-dependent patients is 0.270 units lower than dependent patients and the postoperative recovery index scale score of independent patients is 0.699 units lower than dependent patients (Table 7).

**Table 7:** Examination of factors effective on the care dependency scale score of the postoperative recovery index.

The factors	Standardized coefficient		Unstandardized coefficient	*t*	*p*	95.0% CI	
	B.	Std. mistake	Beta			Lower limit	Upper limit
(constant)	4,886	0.366		13,362	0.000	4,160	5,611
Care dependency scale	−0.032	0.007	−0.461	−4.536	0.000	−0.045	−0.018
semi-dependent/dependent	−0.270	0.110	−0.194	−2,460	0.016	−0.488	−0.052
Independent/dependent	−0.699	0.193	−0.390	−3.625	0.000	−1.082	−0.316

F: 51.854, *p* < 0.001, R^2^: 0.618.

A multivariate linear regression model was conducted with the PORI total score as the dependent variable. Independent predictors included: CDS score, Dependency level (dependent / semi-dependent / independent), Age, sex, education, comorbidity, operated side, postoperative day, and time to mobilization. The model explained 62% of the variance (R^2^ = 0.62, *p* < 0.001). Increasing CDS scores were associated with lower PORI total scores (*β* = −0.031, 95% CI: −0.045 to −0.018, *p* < 0.001). Older age and longer time to mobilization were significantly associated with poorer recovery outcomes. Gender was not significantly related to recovery (*p* = 0.31).

There is a statistically significant negative correlation between age and care dependency scale, and a statistically significant positive correlation between age and postoperative recovery index and psychological symptoms, physical activities, general symptoms, appetite symptoms subscale scores (*p* < 0.05; Table 8).

**Table 8:** Examining the relationship between patients’ age and care dependency scale score and postoperative recovery index score.

Patients’ age		PBB	psychological symptoms	Physical activities	General symptoms	Bowel symptoms	Appetite symptoms	PoRI
Age	*r*	−0.361	0.393	0.352	0.295	0.119	0.299	0.367
	*p*	**0.000**	**0.000**	**0.000**	**0.003**	0.239	**0.003**	**0.000**
	N	one hundred	one hundred	one hundred	one hundred	one hundred	one hundred	one hundred

r, Pearson correlation coefficient. *p < 0.05.

## Discussion

In this section, the findings obtained from the Care Dependency Scale and Postoperative Recovery Index of patients who underwent total knee replacement are discussed under two headings.

### Dıscussıon of the fındıngs of the care dependency scale

When the relevant literature on the care dependency status of the patients was examined; it was seen that the mean care dependency scale scores were between 48.80 and 73.79 in the studies (9–13). In this study, the mean care dependency scale score of 58.43 indicates that the patients participating in the study were partially dependent in accordance with the literature.

In this study, it was found that there was no statistically significant difference between genders in terms of care dependency scale score. When the relevant literature is examined, there are studies in which there is no significant difference between gender and care dependency scale in line with the study findings (14, 15). On the other hand, Caljouw et al. (16), found that women had more care dependency needs than men, Pekince and Aslan (17) found that there was a significant difference between gender and care dependency and female patients were more dependent than male patients, The reason for finding a significant difference according to gender in the study can be interpreted as nurses provide care services equally to both groups without gender discrimination.

When the relationship between marital status and care dependency was examined, it was found that unmarried patients were more dependent than married patients. In the study conducted by Pekince and Aslan (17), it was found that there was a significant relationship between care dependency and marital status and the dependency level of divorced patients was higher than married and single patients. On the other hand, in the study conducted by Düzgün et al. (10), no significant difference was found between care dependency and marital status. In this study, the reason why single patients were more dependent than married patients can be explained by the fact that the social and emotional etc. needs of married patients are met by their spouses.

When the literature examining the relationship between age and care dependency was reviewed; it was found that care dependency increased with age (3, 9–11). However, in the study conducted by Kılıç et al. (15), it was found that there was no significant difference between the care dependency scale and age. With this study, it can be said that the reason for the increase in care dependency as the age of the patients increases is that the diseases, needs and drug use increase as the age of the patients increases in accordance with the literature.

In many studies in the literature, it was found that care dependency increased as the education level of patients decreased (10, 15, 17–19). On the other hand, in the study conducted by Güler et al. (12), no statistically significant difference was found between the mean care dependency scale scores of the patients and their educational status. In this study, the decrease in the level of care dependency of the patients with increasing education level is consistent with the literature, as patients with higher education level are compatible with the treatments applied or they are conscious about their diseases.

In the study, it was concluded that retired patients were more dependent than working patients. In parallel with the result of the study, in the study conducted by Güler et al. (12), it was found that the level of care dependency of patients who were not working in any job was higher than those who were working, and in the study conducted by Türk and Üstün (9), the average care dependency scale score of patients who were civil servants was the highest and the care dependency scale score of patients who were farmers was the lowest, that is, patients who were farmers were more dependent than civil servants. In contrast to these studies, in the study conducted by Düzgün et al. (10), to determine the care dependency levels of patients hospitalized in the chest diseases service of a university hospital, no significance was found between occupational group variables and the total score of the care dependency scale. In this study, the reason why retired patients were found to be more dependent than working patients may be due to the fact that retired patients have comorbid diseases, their social support systems have decreased because their children have grown up, they have economic difficulties, retired patients spend most of their time resting at home and they need more care to fulfil their daily living activities because they are older in average age.

The care dependency scale score of patients with a clinical diagnosis of left TKA was higher than patients with a clinical diagnosis of right TKA, and patients with left TKA surgery were found to be more independent than patients with right TKA surgery. On the other hand, in the study conducted by Düzgün et al. (10), no significance was found between medical diagnosis and total score of the care dependency scale. In this study, the reason why patients with a clinical diagnosis of right TKA were found to be more dependent than patients with a clinical diagnosis of left TKA may be due to the fact that patients with right TKA surgery cannot move their right feet as comfortably as they want, since the right side of the body is mostly used while meeting the physical needs of the patients.

When the studies in the literature examining the relationship between having a chronic disease and patients’ care dependency are examined, there are studies that do not find a significant difference between care dependency and chronic disease (12, 13); on the other hand, there are studies in which the dependency levels of patients with chronic diseases are high (3, 9, 10).

In this study, no significant difference was found between the presence of chronic disease and care dependency. The reason for this result in the study may be that patients with chronic diseases develop a defense mechanism against the difficulties arising from their current diseases and that the presence of chronic disease does not affect the level of dependency because they have adapted to care dependency.

In the study, no statistically significant difference was found between the status of previous surgical operation and care dependency scale score. In the study conducted by Pamuksuz (20) on the subject, when the patients’ history of previous surgical intervention was examined; it was concluded that the care dependency scale scores of patients with a history of surgical intervention were lower than the scale scores of patients who had not undergone surgical intervention before. In this study, the fact that patients with a history of previous surgical intervention did not affect care dependency may be attributed to the fact that care dependency is a factor specific to each surgical intervention due to the different types of surgical interventions that patients undergo.

In the study, it was found that patients with a care dependency scale score on the 4th day of surgery were more independent than patients on the 1st day of surgery. In contrast to the study, in the study conducted by Baksi et al. (11), it was concluded that patients who underwent surgical intervention were more dependent as the total hospitalization period increased. In the study conducted by Aydın and Gürsoy (21), no significant relationship was found between the level of care dependency and total length of hospitalization. In this study, the fact that patients who were on the 4th day of surgery were more independent than patients who were on the 1st day of surgery can be explained by the fact that as time passes, patients can move more easily due to decreased pain and increased participation in daily living activities.

When the time of first mobilization of the patients was examined, patients with mobilization on the first day were more independent than patients with first mobilization time 0–12 h. In contrast to this study, Aygin et al. (22), concluded that delayed mobilization in the postoperative period increased the dependency level of the patient. In this study, the reason why patients who were mobilized later were more independent may be explained by the fact that the severity of pain experienced by the patients decreased over time and caused the patient to feel better.

### Dıscussıon of the fındıngs of the postoperatıve recovery index

The mean postoperative recovery index scale score of the patients was 2.82 ± 0.68. As a result of the study, it was determined that the postoperative recovery level of the patients was very difficult.

As the age of the patients increased, it was determined that the patients had more difficulty in postoperative recovery in the postoperative recovery index and psychological symptoms, physical activities, general symptoms, appetite symptoms sub-dimensions. In parallel with the study, Cengiz and Aygin (8) found that patients experienced more difficulties in general symptoms as the average age of the patients increased. Similarly, in the study conducted by Dığın and Kızılcık Özkan (23) to determine the recovery status of older adults patients after surgery, it was concluded that the advancement of age made recovery difficult. According to the results of the study, it is thought that the increase in physiological and psychological problems with age and the decrease in social support make it difficult to recover in the postoperative period.

No significant difference was found between gender and postoperative recovery index scale and subscale scores. İn contrast to these study, Cengiz and Aygin (8) found that female patients had more difficulty in the recovery process than male patients. In the study conducted by Dığın and Kızılcık Özkan (23) to determine the recovery status of patients with a high average age after surgery, it was concluded that female patients had more difficulty in the recovery process than male patients. In this context, according to the results of the study, it is thought that the mobilization of orthopedic patients undergoing major surgical interventions is painful, affecting the recovery in both male and female patients, and prolonging the process.

It was concluded that single patients had more difficulty in recovery in appetite symptoms than married patients. Similarly, in a study conducted by Dığın and Kızılcık Özkan (23) to determine the recovery status of patients with a high average age after surgery, it was concluded that unmarried patients had more difficulty in postoperative recovery than married patients. Again, Ali et al. (24), conducted a study to determine the quality of life of patients with maxillectomy and found that married patients had a better quality of life. In line with this information; it is seen that the result that emotional and social needs can be met more in the presence of a spouse and postoperative recovery is easier for married patients is in parallel with the literature.

In the study, patients with primary school graduates or illiterate patients had more difficulty in recovery according to the total score obtained from the postoperative recovery index than patients with high school graduates. It can be thought that the data obtained from this study are similar to the literature and that this situation contributes to the easier recovery of people in the postoperative period, as the level of education increases, people question more research and are open to learning and gain awareness.

In the study, it was determined that patients who were not working or retired had more difficulty in recovery than working patients according to the total score obtained from the postoperative recovery index. According to the results of the study, it may be due to the fact that patients who do not work in a job listen to themselves more because they do not have any occupation and perceive all kinds of symptoms of the disease more.

Patients with chronic disease had moderate difficulty in recovery in general symptoms compared to patients without chronic disease. In the study conducted by Evkaya (25). although there was no significant correlation in terms of postoperative recovery index general symptoms sub-dimension score in the case of patients having chronic diseases, in the same study, it was concluded that there was a decrease in the recovery status of patients as the number of chronic diseases in patients increased numerically in terms of postoperative recovery index physical activities sub-dimension. It was observed that our research findings were similar to the literature. In this context, it can be said that the presence of chronic diseases in patients negatively affects the quality of recovery by causing prolonged wound healing, increased postoperative complications and negative effects on the independent performance of daily living activities (8, 26).

In the study, it was found that patients who had undergone surgery before had moderate difficulty in recovery in general symptoms and bowel symptoms compared to patients who had not undergone surgery before. In the study conducted by Cengiz and Aygin (8) it was concluded that patients who had undergone previous surgical intervention had more difficulty in postoperative recovery, and it was concluded that the mean scores in the general symptoms sub-dimension of patients who had undergone previous surgical intervention were statistically significant and the scores were higher than patients who had not undergone previous surgical operation. In line with the literature, it is thought that the negativities related to the hospital and previous surgeries and the number of previous surgeries may negatively affect the recovery status of the patients.

Patients evaluated on postoperative days 1–3 showed lower recovery scores in physical activity, appetite symptoms, and total PORI compared with those assessed on postoperative day 4 or later. These findings indicate an association between earlier postoperative assessment and greater recovery difficulty, rather than a causal effect. In contrast to this study, Evkaya (25) concluded that most of the patients with positive recovery scores had hospitalization periods of less than 1 h and in the range of 1–24 h. In this study, the fact that the recovery status of the patients was positively affected as the hospitalization period was prolonged may be explained by the fact that the pain of the patients who underwent major orthopedic surgery decreased over time and returned to their daily life activities and this situation contributed positively to patient recovery.

İt was determined that patients with a first mobilization time of 0–12 h had extreme difficulty in recovery in the physical activities sub-dimension compared to patients with a first mobilization time of 1 day. In this study, the fact that the patients had difficulty in recovery as a result of early mobilization may have been due to the type of surgical intervention due to major orthopedic surgery.

Although this study provides valuable insights into the relationship between care dependency and postoperative recovery after total knee arthroplasty, certain limitations should be acknowledged. The study did not include data on potential confounding factors such as preoperative physical condition, disease severity, functional status, quality of life, and psychosocial characteristics. These factors may influence both nursing dependency and postoperative recovery, and their absence should be considered when interpreting the results. Additionally, it should be noted that this study is cross-sectional in design, and therefore only associations between care dependency and postoperative recovery can be identified; causal relationships cannot be established. Future prospective cohort or interventional studies are recommended to clarify these causal pathways and better understand the mechanisms underlying nursing dependency after total knee arthroplasty.

## Conclusion

As a result of this study conducted to determine the effect of care dependency levels on recovery in patients undergoing total knee replacement; care dependency levels in patients undergoing total knee replacement affect the recovery of patients. In the study conducted to determine the effect of care dependency levels on recovery in patients undergoing total knee replacement, recommendations for providing better quality nursing care to patients:

- Nurses should periodically evaluate the pain, independence and fear of movement levels of patients with total knee replacement surgery and plan the necessary nursing care according to these results,
- While planning the care of the patients, nurses should take into account the individualized care perceptions of the patients and the socio-demographic and clinical characteristics that affect their well-being.
- Providing education and counseling services regarding the surgical process by nurses after the decision to operate on patients,
- Implementation of physical therapy programs to facilitate the adaptation of patients with total knee replacement surgery to activities of daily living in the postoperative period and to minimize the fears of patients about moving,
- While the study was conducted in an inpatient setting with significant nursing involvement, the findings can inform outpatient care models as well. For instance, training non-nurse caregivers or implementing remote monitoring systems could support recovery in settings where nursing care is less accessible. Tailored discharge planning and caregiver education should be prioritized to ensure continuity of care and effective recovery support.
- The results of this study should be interpreted within the context of the Turkish healthcare system, where nursing care plays a significant role in inpatient and postoperative settings. In outpatient-focused systems like the United States, where non-nurse caregivers often take the lead post-discharge, these findings highlight the need for enhanced caregiver support and education to address care dependency effectively.
- This study provides valuable insights into the relationship between care dependency and postoperative recovery in TKA patients. Future research should focus on evaluating specific interventions that address care dependency, particularly in outpatient settings. Additionally, exploring culturally tailored strategies could improve the generalizability of these findings across diverse healthcare systems.

## Data Availability

The original contributions presented in the study are included in the article/supplementary material. Further inquiries can be directed to the corresponding author.
